# Effect of surface preparation methods on the shear bond strength of universal and self-etch adhesive systems: an in vitro study

**DOI:** 10.1016/j.jobcr.2026.101408

**Published:** 2026-01-19

**Authors:** Shiwangi Verma, Ravishankar Suman, Rajendra Goud, Snigdho Das, Srikant Gollapudi, Samapika Routray

**Affiliations:** a Department of Dentistry, All India Institute of Medical Sciences, Bhubaneswar, Odisha, India; b School of Minerals, Metallurgical and Materials Engineering, Indian Institute of Technology, Bhubaneswar, Odisha, India; c Department of Dentistry, Ramakrishna Sarada Mission Matri Bhavan Hospital, Kolkata, West Bengal, India

**Keywords:** Adhesives, Air abrasion, Dental bonding, Dental stress analysis, Dentin, Shear strength

## Abstract

**Objectives:**

To assess the impact of three smear layer preparation methods on shear bond strength (SBS) to enamel and dentin, using two universal adhesives (Tetric N-Bond Universal, 3M Single Bond Universal) and one two-step self-etch adhesive (Kuraray Clearfil SE Bond).

**Methods:**

Ninety extracted premolars were assigned to enamel and dentin groups (n = 45 each) and prepared with coarse grit (120), fine grit (600) silicon carbide paper, or air abrasion (Aluminium Oxide for enamel, Sylc for dentin). Surfaces were bonded with one of the adhesives and restored with composite. Enamel was treated in an etch-and-rinse mode, dentin in self-etch mode. SBS was tested using a universal testing machine. Failure surfaces were examined under SEM to assess fracture morphology and EDS to determine elemental composition. Data were analyzed using two-way ANOVA/Tukey's post-hoc test and Weibull statistics (P ≤ 0.05).

**Results:**

In enamel, SBS generally increased from coarse to fine to air abrasion. Kuraray Clearfil SE Bond yielded the highest values, particularly with air abrasion (9.11 ± 0.51 MPa). In dentin, fine grit achieved the highest SBS across all adhesives, followed by air abrasion; coarse grit showed the lowest. Kuraray consistently outperformed the universal adhesives in most dentin conditions. SEM/EDS revealed predominantly adhesive failures; occasional cohesive failures in dentin (Ivoclar) and enamel (Kuraray) corresponded with higher SBS and Weibull reliability.

**Conclusion:**

Both surface preparation and adhesive type significantly influence SBS. Kuraray showed superior performance and reliability, while fine grit and air abrasion enhanced bonding efficacy. SEM/EDS findings corroborated the mechanical results.

## Highlights

• Surface preparation method and adhesive type significantly influence SBS.
• Fine grit finishing and air abrasion improved bonding efficacy.
• Chemically compatible adhesives with proper protocols improve predictability and durability of restorations.
• For long lasting adhesion, match the material with the appropriate clinical protocol.

## Graphical abstract

**Figure:**
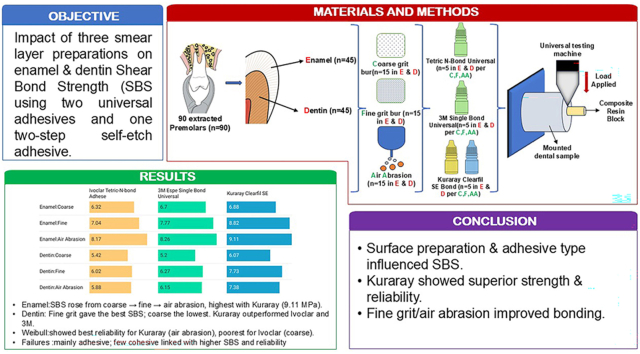

## 1 Introduction

Longevity of restorations depends greatly on the adhesive interface between tooth and restorative material. Universal adhesives, available as all-in-one systems, have simplified procedures but vary in pH and bonding effectiveness depending on the substrate and preparation method.^1^ During cavity preparation, the smear layer formed on enamel or dentin can hinder adhesive infiltration and compromise bonding, particularly with self-etch systems.^2^ Surface modification strategies such as silicon carbide abrasion and air abrasion aim to optimize this interface. Air abrasion using alumina or bioactive glass has been reported to produce thinner smear layers, potentially improving resin penetration and reducing microleakage.^3^ In recent years, Sylc, a prophylactic powder containing NovaMin, has been introduced, releasing calcium and phosphate ions to form a stable apatite layer on treated surfaces.^4^

Despite such developments, the influence of smear layer variations on modern universal adhesives remains inadequately studied. Kuraray Clearfil SE Bond is widely regarded as the benchmark two-step self-etch adhesive due to the presence of 10-MDP and long-term durability.^5^ Comparisons with newer single-step adhesives such as Tetric N-Bond Universal and 3M Single Bond Universal, which claim broad applicability, are clinically relevant. Although adhesive systems are often evaluated independently, their clinical performance is strongly influenced by surface preparation protocols and application strategies. Therefore, the present study evaluates shear bond strength as a combined outcome of adhesive type and surface pretreatment technique, reflecting a more technique-sensitive and clinically relevant bonding approach.

## 2 Material and methods

The study protocols were approved by the Institutional Ethics Committee (IEC No: T/EMF/Dentistry/21/81).

Study materials- Adhesive was used in self-etch mode for the dentinal surface and etch and rinse approach for the enamel surface.

**Fig. 1:**
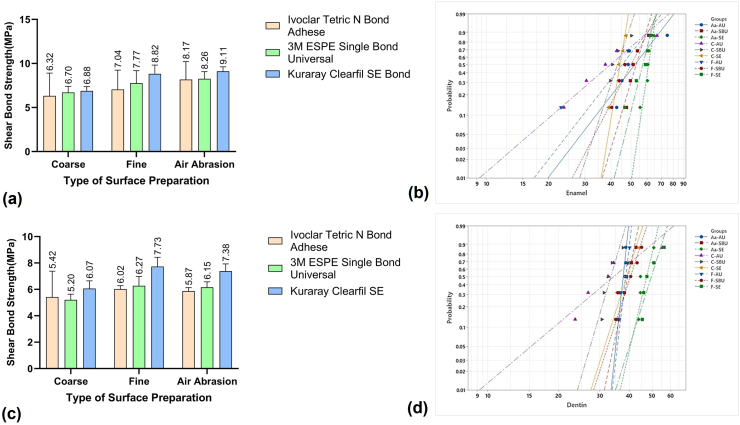
(a) Mean ± Standard deviation (SD) of shear bond strength (SBS) for the three adhesive systems according to the type of surface preparation for enamel specimens. (b) Weibull analysis for SBS data of enamel specimens, with fitting curves based on Pf = 1 – exp[–(σ/σ_0_)ᵐ]. (c) Mean ± SD of SBS for the three adhesive systems according to the type of surface preparation for dentin specimens. (d) Weibull analysis for SBS data of dentin specimens, with fitting curves based on Pf = 1 – exp[–(σ/σ_0_)ᵐ]. AU: Ivoclar Tetric-N-bond Adhese Universal; SBU: 3M Espe Single Bond Universal; SE: Kuraray Clearfil SE; C: Coarse; F: Fine; AA: Air Abrasion. ↑ Steepness of the curve indicates ↑ Weibull's modulus and hence ↑ reliability.

Specimen preparation- Ninety freshly extracted young premolar teeth for orthodontic purposes were stored in 10 % formalin solution and used within 6 months. The teeth were sectioned at a 3 mm distance from the cemento-enamel junction (CEJ) with a diamond disc mounted on a slow-speed straight handpiece (NSK; Nakanishi, Japan) under water spray. The coronal part was mounted in a self-cure acrylic cylinder by using silastic mold (Fig. 1). The prepared test specimen was embedded in water to dissipate heat. The prepared test specimen was divided randomly into two groups(*n* = 45):

1. Enamel group
2. Dentin group.

The coronal buccal surface was ground flat with the help of a stone trimmer under water cooling. In the enamel group surface was examined under magnification to ensure the ground enamel surface was intact, whereas in the dentin group surface was inspected to confirm the pulp was not exposed. Teeth were divided in to 3 subgroups(*n* = 15) according to surface preparation:

Group I– Wet coarse silicon carbide paper (120 grit) was used at low speed with the handpiece running at 10000 rpm for 30 s in a circular motion.
Group II– Wet Fine silicon carbide paper (600 grit) was used.
Group III - Enamel-surface treatment done with an air abrasion unit (Aqua Care Twin, Velopex) with the 29μ Aluminium Oxide for 10 s at 7 bar/100 psi, whereas dentin-surface treatment was carried out with the help of Sylc for 10 s.

Teeth specimens were washed under distilled water with an air-water spray for 20 s and air-dried for 20 s.

Bonding procedure –Each surface preparation group was again subdivided into three adhesive subgroups:

a) TNBU (Ivoclar Vivadent; Schaan, Liechtenstein)
b) SBU (3M Oral Care; St Paul, MN, USA)
c) CSB (Kuraray Noritake; Okayama, Japan)

The enamel surface was conditioned with 37 % phosphoric acid for 15 s and washed with distilled water and dried with the blot drying technique. The prepared specimen was secured in the clamp with a polytetrafluoroethylene mold with a cylindrical cavity of 2.4 mm in diameter and 4 mm in height. The composite restoration (Tetric N-Ceram, Ivoclar Vivadent, Schaan, Liechtenstein) was filled in two increments of 2 mm and cured for 40 s according to the manufacturer's instructions with a light curing unit (Bluephase N, Ivoclar Vivadent, Schaan, Liechtenstein) having a minimum output energy of 500 mW/cm^2^. The light-cured restoration was allowed to self-cure for 1 min. The bonded assembly was removed from the clamp and immersed in water at 37 °C for 24 h before SBS evaluation.

### 2.1 SBS test

The specimen was loaded to failure utilizing a universal testing machine (Zwick Roell, Germany) mounted with a 5 kN load cell and at a crosshead travel rate of 1 mm/min. A custom-made shearing fixture was used to apply load on the bonding assembly proximately to flat ground tooth surfaces. SBS (MPa)was determined from the peak load at failure divided by the bonded surface area.

### 2.2 Fractographic and elemental analysis

After conducting SBS testing, representative specimens that failed from each group were analyzed using a field emission scanning electron microscope (SEM) (ZEISS, Oberkochen, Germany) to investigate fracture morphology and failure modes. In conjunction with SEM, energy dispersive spectroscopy (EDS) [XL30, FEI, Hillsboro, USA] was utilized to ascertain the elemental composition of the failed surfaces, aiding in the differentiation between adhesive and cohesive failures by examining the presence of substrate-specific elements in contrast to adhesive resin components.

## 3 Statistical analysis

Normality of the data was assessed using the Shapiro–Wilk test. Following confirmation of normal distribution, two-way Analysis of Variance (ANOVA) was implemented to analyse mean SBS variations across three adhesive systems under differing surface preparations. Post hoc Tukey's test was used for pairwise comparisons. A *P*-value of ≤0.05 was considered statistically significant. Statistical analysis was conducted using GraphPad Prism for Windows, Version 10.1.2 (GraphPad Software, La Jolla, California, USA).

### 3.1 Weibull analysis

Weibull's analysis begins by ranking the samples in ascending/descending order. The values which are plotted along the horizontal axis (x-axis) of the graph are obtained as natural logarithms of the SBS.

For each specimen (*n*), the probability of failure (Pf) was determined by the following equation

Pf = (i-0.5)/*n*

where *i* denotes the specimen's rank in ascending order of bond strength values (1 = weakest, *n* = strongest). The vertical (y) axis represents the double natural logarithm of 1/(1 − Pf). The plotted points are fitted with a straight line using linear regression, and the slope of this line denotes the Weibull modulus (*m*).^6^

Maximum likelihood estimation was used for the two-parameter Weibull distribution, including the Weibull's modulus(*m*) and the scale parameter (characteristic SBS) to interpret the reliability of SBS (Minitab Software V.18.1, State College, PA, USA).

A statistical significance threshold of *P* ≤ 0.05 was used throughout.

## 4 Results

### 4.1 Enamel

All three adhesives demonstrated a general trend of increasing shear bond strength (SBS) from coarse to fine to air abrasion (Table 1, Fig. 1a). For Ivoclar TNBU, the mean values rose from 6.32 ± 2.59 MPa with coarse preparation to 7.04 ± 2.20 MPa with fine grit and 8.17 ± 2.05 MPa with air abrasion, although these differences were not statistically significant (*P* > 0.73). The 3M SBU group also showed a progressive increase from 6.70 ± 0.705 MPa (coarse) to 7.77 ± 1.41 MPa (fine) and 8.26 ± 0.807 MPa (air abrasion), with a significant difference between coarse and air abrasion (*P* = 0.0361). Kuraray CSB displayed the most consistent performance, with SBS values rising from 6.88 ± 0.491 MPa (coarse) to 8.82 ± 0.998 MPa (fine; *P* = 0.0252) and 9.11 ± 0.511 MPa (air abrasion; *P* = 0.0003). While no significant differences were found between the adhesives in coarse and fine groups, CSB showed borderline superiority under air abrasion conditions. Weibull analysis (Table 2, Fig. 1b) further highlighted this trend, with CSB under air abrasion demonstrating the highest modulus and characteristic bond strength (m = 22.38, σ_0_ = 61.95 MPa), whereas Ivoclar TNBU under coarse grit yielded the weakest reliability. These findings led to a partial rejection of the first null hypothesis, as surface preparation significantly influenced enamel SBS, while adhesive type was less decisive.

**Table 1:** Mean (Standard deviation) of the SBS for the three adhesive systems according to the type of surface preparation for enamel and dentin samples respectively.

	Type of surface preparation	Ivoclar Tetric-N-bond Adhese	3M Espe Single Bond Universal	Kuraray Clearfil SE
**Enamel**	**Coarse**	6.32(2.59) a1A1	6.7(0.705) a1A1	6.88(0.491) a1A1
	**Fine**	7.04(2.2) a1A1	7.77(1.41) a1b1A1	8.82(0.998) b1A1
	**Air Abrasion**	8.17(2.05) a1A1	8.26(0.807) b1A1	9.11(0.511) b1A1
**Dentin**	**Coarse**	5.42(1.96) a2A2	5.2(0.436) a2A2	6.07(0.58) a2A2
	**Fine**	6.02(0.259) a2A2	6.27(0.7) b2A2B2	7.73(0.703) b2B2
	**Air Abrasion**	5.88(0.257) a2A2	6.15(0.424) b2A2	7.38(0.554) b2B2

Different lower-case letters in superscript denote a statistically significant difference within the groups/in a row (type of surface preparation) analyzed by *post-hoc* Tukey's test.

Different uppercase letters in superscript denote a statistically significant difference between the groups/in a column (adhesive systems) analyzed by *post-hoc* Tukey's test.

1: Enamel specimens; 2: Dentin specimens.

**Table 2:** Weibull's modulus(*m*) and characteristic strength(σ_0_)along with their respective confidence intervals for the three adhesive systems according to the type of surface preparation for enamel and dentin samples respectively.

Adhesives	Type of Preparation	Enamel		Dentin	
		*m*(95 %CI)	σ_0_ (95 %CI)	*m*(95 %CI)	σ_0_ (95 %CI)
**Ivoclar Tetric N Bond Adhese**	Coarse	2.9(1–4.8) a1	45.29(30.75–59.84) a1b1	3.21(1.15–5.27) a2	38.59(27.38–49.8) a2c2
	Fine	4.31(1.19–7.43) a1	49.4(38.89–59.91) a1b1	32.03(8.88–55.18) a2	39.1(37.97–40.23) a2c2
	Abrasion	4.36(1.63–7.1) a1	56.99(44.78–69.21) a1b1	35.69(8.35–63.02) a2	38.11(37.13–39.09) a2c2
**3M ESPE Single Bond Universal**	Coarse	10.02(3.66–16.37) a1	44.73(40.56–48.9) a1	12.9(4.61–21.18) a2	34.4(31.91–36.89) a2
	Fine	6.46(2.19–10.73) a1	53.11(45.46–60.77) a1b1	11.73(3.42–20.05) a2	41.8(38.5–45.1)a2b2bc2
	Abrasion	11.11(3.98–18.25) a1	54.93(50.32–59.54) a1b1	18.18(5.79–30.57) a2	40.37(38.31–42.43) a2c2
**Kuraray Clearfil SE**	Coarse	20.12(5.7–34.54) a1	45.15(43.08–47.22) a1b1	12.02(4.11–19.93) a2	40.28(37.16–43.4) a2c2
	Fine	13.26(3.32–23.2) a1	58.74(54.68–62.8)	12.02(4.25–19.8) a2	51.27(47.29–55.25) b2c2
	Abrasion	22.38(7.37–37.39) a1	61.95(59.4–64.5) b1	16.24(4.94–27.54) a2	48.59(45.8–51.37) c2

*m:* Weibull's modulus.

σ_0:_ characteristic strength (scale parameter).

CI: Confidence intervals.

Different lower-case letters in subscripts denote a statistically significant difference between the groups and the subgroups/in a column for each shape and scale parameter for enamel and dentin, respectively.

### 4.2 Dentin

In dentin, Ivoclar TNBU showed minimal variation across preparations, with values ranging from 5.42 to 6.02 MPa and no significant differences (*P* > 0.99) (Table 1, Fig. 1c). For 3M SBU, air abrasion significantly improved SBS compared to coarse (6.15 ± 0.42 vs. 5.20 ± 0.43 MPa; *P* = 0.0246), while the difference between coarse and fine was not significant (*P* = 0.0728). Kuraray CSB again showed superior performance, with SBS increasing significantly from 6.07 ± 0.58 MPa (coarse) to 7.73 ± 0.703 MPa (fine; *P* = 0.0114) and 7.38 ± 0.554 MPa (air abrasion; *P* = 0.0194). Between-group comparisons confirmed that CSB performed significantly better than Ivoclar and 3M in both fine and air abrasion groups (*P* = 0.0153–0.05). Weibull reliability analysis (Table 2, Fig. 1d) indicated that Ivoclar with fine grit and air abrasion achieved acceptable reliability (m ≈ 32–36), but coarse grit produced poor values (m = 3.21). The highest characteristic strength was recorded for CSB under fine preparation (σ_0_ = 51.27 MPa), which was significantly greater than 3M SBU under coarse preparation (σ_0_ = 34.4 MPa; *P* = 0.003). These outcomes resulted in rejection of the second null hypothesis, as both surface preparation and adhesive type significantly influenced dentin SBS.

## 5 Fracture mode analysis

**Fig. 2:**
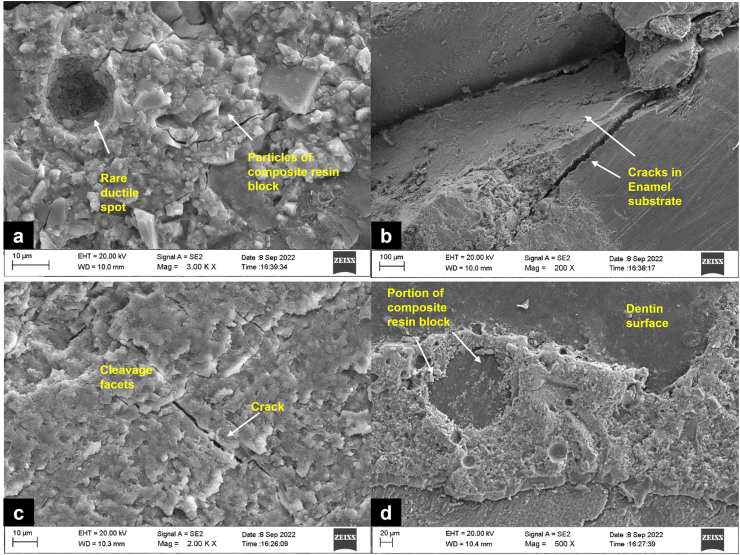
Scanning electron microscopy (SEM) images of fractured specimens after shear bond strength testing: (a) rare ductile region with embedded particles of composite resin block in enamel ( × 3000), (b) crack propagation within the enamel substrate following fracture ( × 200), (c) morphology of dentin showing cleavage facets and crack formation ( × 2000), (d) adhesive failure with remnants of composite resin block attached to the dentin surface ( × 500).

Fractographic analysis corroborated these findings. Most failures were adhesive across all groups; however, cohesive failures appeared only in dentin specimens bonded with Ivoclar TNBU under air abrasion and in enamel specimens bonded with Kuraray CSB under air abrasion. These cohesive events aligned with higher SBS and Weibull reliability in those groups. In contrast, 3M SBU showed exclusively adhesive failures, supporting its relatively weaker bonding profile (Fig. 2).

## 6 Discussion

The rise in the usage of direct tooth-colored restorations has led to research on improving the effectiveness and longevity of the resin-dentin bonds.^3^ Universal adhesive systems have been introduced in recent years and have rapidly gained attention due to their flexible applicability and low technique sensitivity.^7^ However, concerns regarding their bond strength while bonding to different smear layers persist.^2^ It is already proven that single-bottle self-etching adhesives have a relatively lower pH and are more hydrophilic than the multi-bottle variants, which makes them prone to nanoleakage due to plasticization of the resin matrix and ultimately poorer bond strength.^8^, ^9^, ^10^, ^11^ Thus, this study was carried out to assess to evaluate the effect of different smear layer patterns formed by preparing the tooth with a coarse grit bur, fine grit bur, and air abrasion, respectively in relation to SBS of enamel and dentin, utilizing one sixth-generation and two seventh-generation universal adhesives.

It was observed that specimens prepared using air abrasion had the highest SBS than the specimens prepared with a fine-grit and coarse-grit bur, respectively, in enamel. These findings were reinforced by Kui et al.^12^ CSB by Kuraray has been considered the “gold standard” in adhesive dentistry owing to its superior strength and stability.^13^

The findings concorded with the results of the present study, where Kuraray CSB, a sixth-generation bonding agent, exhibited higher SBS values compared to other groups, which belonged to the seventh generation of the dental adhesives. It has been proposed that higher SBS by two-step adhesives is due to the low concentration of solvents and low hydrophilicity.^14^ However, no noteworthy differences were found between the three groups, which correlated with the findings of Beltrami et al., reporting a higher bond strength of Kuraray CSB compared to seventh-generation adhesives, but without a significant difference. The usage of high-density fillers in Kuraray CSB enhances mechanical adhesion by filling the microporosities created on the pretreated enamel.^15^ Based on the results obtained in the present study, the first null hypothesis stands partially rejected as there was an effect of only the type of surface preparations on the SBS of enamel found for the study groups.

In the dentin specimens, like enamel, preparation with a coarser-grit bur exhibited significantly lower SBS values than fine-grit bur for all three study groups. However, contrary to the findings in the enamel specimens, models prepared with air abrasion had lower SBS than those prepared with a finer-grit bur for all three groups, but higher than those prepared with a coarser bur. Lesser SBS values for the air-abrasion specimens can be attributed to the formation of surface irregularities, which compromise the etching ability of the adhesives.^16^ The results closely corroborate the findings of Anja et al., who also failed to demonstrate any effect on bond strength after pre-treating the dentin with air abrasion.^17^ Also, the choice of material may have a role, as in a previous study, air abrasion with 50 μm aluminum oxide considerably improved the bond strength. In the current study, the use of bioactive glass may have attenuated the infiltration of the adhesive, due to occlusion of the dentinal tubules, although the reason is purely hypothetical and needs further investigation. Nonetheless, the Kuraray CSB group exhibited the highest SBS amongst all the study groups for all types of surface preparations, which corroborates the findings of Salvio et al.^18^ and Levartovsky et al.^19^ Tay et al.^20^ observed that Kuraray CSB can penetrate the partially demineralized smear layer and form a hybrid layer. Another study reported greater porosities in the inter-tubular dentin with a superfine-grit bur, creating more channels, facilitating better adhesive monomer penetration.^21^ The results from the dentin specimens led to the complete rejection of the second null hypothesis, as a significant effect of both the dental adhesive and the type of surface preparation was found to affect the SBS.

In the present study, Weibull statistics were employed as it is regarded as a valid method for assessing a material's reliability. It contributes to retrieving the material's dependability and the chance of failure corresponding to a specified stress level.^22^ McCabe et al. reported that a Weibull modulus value of at least 10 is necessary for a test to be acceptable for multicentre testing.^23^ In the current study, except for a few samples in the Ivoclar TNBU and the 3M SBU group, which demonstrated a Weibull Modulus of 10 or above, thus ensuring that the materials were tested with the most suitable test design. Used to reflect the variability in the material's strength, a higher*,* even if associated with slightly lower bond strength, is often regarded as more desirable than a lower *m* that corresponds to increased bond strength.^23^ An increased m value also reflects a better-suited test design for assessing and comparing material strength.^22^ In the present study, a higher *m* was found in the Kuraray CSB group among the enamel specimens and the least in the Ivoclar TNBU group, whereas interestingly, among the dentin specimens, a higher *m* was evident in the fine and air abrasion-treated Ivoclar specimens. However, the evidence was too weak to prove the statements.

These differences in bonding performance were further reflected in the failure mode analysis. The predominance of adhesive failures across groups highlights the relative weakness at the tooth-restoration interface. However, cohesive failures were observed in two dentin specimens from the Ivoclar group and one enamel specimen from the Kuraray CSB group, both treated with air abrasion. The occurrence of such failures suggests a stronger bond at the adhesive interface, occasionally exceeding the cohesive strength of the composite itself.^24^ These findings align with the higher SBS and Weibull reliability parameters noted for these groups, especially Kuraray CSB, which has consistently demonstrated deeper hybrid layer formation and enhanced mechanical interlocking due to its two-step formulation and filler content. In contrast, the complete absence of cohesive or mixed failures in the 3M SBU group underscores a relatively weaker or more brittle bonding pattern, corroborating its lower comparative performance in certain conditions.

In addition to these findings, the present study has several methodological strengths. These include the simultaneous evaluation of multiple surface preparation techniques and adhesive systems on both enamel and dentin substrates. The inclusion of a well-established two-step self-etch adhesive alongside contemporary universal adhesives allows meaningful clinical comparison. Additionally, the combined use of Weibull statistics and fractographic SEM–EDS analysis enhances the reliability and interpretability of bond strength outcomes.

However, the authors cannot disregard the limitation of a smaller sample size, which restricts the reliability of the Weibull analysis, and therefore, the results obtained are purely indicative. Also, all the clinical conditions could not be replicated in laboratory test design, such as pulpal pressure, thermal cycling, pH cycling, and cyclic loading, to simulate the physiological conditions in the oral cavity, including repeated expansion and contraction due to temperature variations and changes in pH due to ingestion and production of acidic chemicals.^25^ Thus, further studies are required utilizing more aggressive tests to study the effect of temperature and other environmental conditions of the oral cavity, over a prolonged period, to verify the sustained impact of the surface preparations and adhesives over time on the SBS in enamel and dentin, respectively.

## 7 Conclusion

In light of the constraints of this in vitro study, the results emphasize that the meticulous choice of surface preparation methods and adhesive systems can significantly impact the reliability and quality of resin-tooth bonding. Utilizing adhesives that demonstrate a verified chemical affinity to dental substrates, in conjunction with preparation techniques that improve substrate conditions, may increase the predictability and durability of restorations in clinical settings. These findings highlight the necessity of aligning material characteristics with suitable operational protocols to ensure lasting adhesive performance.

## Patient's/Guardian's consent

The authors declare that there was no role for consent in the work reported in this paper.

## Ethical clearance

The authors declare the study protocols were approved by the Institutional Ethics Committee (IEC No: T/EMF/Dentistry/21/81).

## Sources of funding

The authors declare there was no external source of funding involved in this study.

## Declaration of competing interest

The authors declare that they have no known competing financial interests, personal relationships, or external funding sources that could have appeared to influence the work reported in this paper.

## List of Abbreviations

•**SBS** –: Shear bond strength
•**TNBU** –: Tetric N-Bond Universal
•**SBU** –: Single Bond Universal
•**CSB** –: Clearfil SE Bond
•**SEM** –: Scanning electron microscopy
•**EDS** –: Energy-dispersive spectroscopy
•**ANOVA** –: Analysis of variance
•**CI** –: Confidence interval
•**MDP** –: 10-Methacryloyloxydecyl dihydrogen phosphate
•**IEC** –: Institutional Ethics Committee
•**CEJ** –: Cemento-enamel junction
•**AA** –: Air abrasion
